# Diagnosis and Treatment of Acute Heart Failure: A Retrospective Observational Study and Medical Audit

**DOI:** 10.3390/jcm13195951

**Published:** 2024-10-07

**Authors:** Justas Suchina, Giorgia Lüthi-Corridori, Fabienne Jaun, Jörg D. Leuppi, Maria Boesing

**Affiliations:** 1 University Institute of Internal Medicine, Cantonal Hospital Baselland, CH-4410 Liestal, Switzerland; 2 Faculty of Medicine, University of Basel, CH-4056 Basel, Switzerland

**Keywords:** acute heart failure, medical audit, Switzerland

## Abstract

**Background**: Acute Heart Failure (AHF) is a leading cause of hospitalizations and remains a significant socioeconomic burden. Despite advances in medical care, mortality and rehospitalization rates remain high. Previous AHF audits have revealed regional differences and a poor adherence to guidelines. This study aimed to assess guideline adherence in a public teaching hospital to identify areas for improvement. **Methods**: This retrospective observational study examined clinical routine data of patients hospitalized for AHF at a Swiss public teaching hospital between 2018 and 2019. AHF management was evaluated against the relevant guidelines of the European Society of Cardiology. **Results**: The study included 760 AHF cases of 726 patients (median age 84 years, range 45–101, 50% female). NT-pro-BNP levels were measured in 92% of the cases. Electrocardiography was performed in 95% and chest X-rays in 90% of cases. Echocardiography was conducted in 54% of all cases and in 63% of newly diagnosed AHF cases. Intravenous furosemide was initiated in 76%. In the subgroup of cases with reduced ejection fraction (HFrEF), 86% were discharged with beta-blockers and 69% with angiotensin-converting enzyme inhibitors or angiotensin II receptor blockers. Among cases with left ventricular ejection fraction ≤ 35%, mineralocorticoid receptor antagonists were prescribed in 55%. **Conclusions**: We observed generally good adherence to guideline recommendations. However, several improvements are needed in initial assessment and documentation, diagnostic procedures such as echocardiography, discharge medication, and lifestyle recommendations. Compared to other studies, our diagnostic workup was more aligned with guidelines, the use of intravenous diuretics was similar, and the duration of hospital stay and mortality rates were comparable.

## 1. Introduction

Heart failure (HF) is a major contributor to both morbidity and mortality worldwide, resulting in substantial healthcare costs and placing a significant burden on both individuals and society [1]. In Switzerland, the number of patients with heart failure is reaching approximately 200,000 with an annual incidence of 2.3 patients per 1000 [2,3]. While an improved understanding of pathophysiology and advancements in treatment for chronic heart failure have significantly reduced the 1-year mortality rate, the outcomes for patients admitted with acute heart failure (AHF) have largely remained unchanged [4]. Most published registries reveal that in-hospital mortality rates for AHF range between 4% and 7%, with a median hospital stay lasting from 4 to 11 days [5]. Other studies showed alarmingly high rates of death or recurrent hospitalization within months, approaching up to 50% [6].

In contrast to the management of chronic HF, no notable improvement in clinical outcomes has been observed in the management of AFH [7]. Furthermore, several studies have identified significant differences between guideline recommendations and patient management for AHF [8]. To change this, health experts, in addition to long-term goals such as the development of new therapeutic methods, emphasized guideline-compliant quality assurance of existing treatment processes [9]. Clinical audits began as a way for clinicians to evaluate medical care against evidence-based best practices, aiming to motivate improvements by identifying care gaps [10]. In the continued presence of a quality gap between actual provided care and medical evidence, health institutions employ quality (QI) strategies, which use systematic, data-driven approaches to monitor and evaluate the quality of hospital care [11,12].

To contribute valid data, we conducted a retrospective study of the AHF management at a Swiss public teaching hospital. The main goal of this study was to assess adherence to existing guidelines. In the second step, the results were compared with other existing audits and registries, to reevaluate where there is potential for improvement.

## 2. Materials and Methods

### 2.1. Study Design

In this retrospective, single-center observational study, we examined clinical routine data of patients who presented with AHF at one of the three sites—Liestal, Bruderholz, or Laufen—of the Cantonal Hospital of Baselland, Switzerland during 2018 and 2019. Cases of the patients who were hospitalized multiple times for AHF within the study period (*n* = 34) were analyzed as separate observations. The study was approved by the Ethical Committee of Northwestern and Central Switzerland (Project-ID 2022-00919).

### 2.2. Study Population

All cases of patients aged 18 and older who were treated and hospitalized for at least one night for AHF as their primary diagnosis were included in the study. Cases were extracted from the internal hospital data base using relevant ICD-10 codes, as listed in the Supplementary Material (Table S1). Cases of patients who had declined the general research consent were excluded from the analysis.

### 2.3. Data Collection and Analysis

Patient demographic information was extracted from the hospital’s controlling data. All other variables including vital signs, laboratory values, symptoms, diagnostics, treatment, medical history and patient outcome were manually collected from electronic patient records. The collected data were entered into a REDCap^®^ database. Data analysis, including the determination of mean, median, standard deviation, range and interquartile range, was performed using REDCap^®^ Software version 14.3.9.

### 2.4. Guidelines

The study findings were benchmarked against the 2016 European Society of Cardiology (ESC) guidelines for the diagnosis and treatment of acute and chronic heart failure [13].

Primary emphasis was placed on Class I recommendations regarding anamnesis, diagnostics and therapy in AHF patients, which denote evidence that a specific treatment or procedure is beneficial, useful, and effective. Furthermore, other interventions and discharge planning were analyzed. Adherence to the following guideline recommendations was assessed in this study:

Diagnostics of AHF [13]:

- During admission, for all patients with suspected AHF, the following diagnostic tests are recommended:
  - Standard non-invasive monitoring of heart rate, rhythm, respiratory rate, oxygen saturation and blood pressure;
  - A measurement of plasma natriuretic peptide level NT-proBNP;
  - The following laboratory assessments in the blood: cardiac troponins, blood urea nitrogen (BUN) (or urea), creatinine, electrolytes (sodium, potassium), glucose, complete blood count, liver function tests and thyroid stimulating hormone (TSH);
  - 12-lead ECG and chest X-ray;
  - Echocardiography is recommended immediately in haemodynamically unstable AHF patients and within 48 h when cardiac structure and function are either not known or may have changed since previous studies.

Therapy of AHF [13]:

- Intravenous loop diuretics are recommended for all patients with AHF admitted with signs/symptoms of fluid overload to improve symptoms.

Discharge planning [13]:

- An angiotensin-converting enzyme inhibitor (ACE-I) is recommended, in addition to a beta-blocker, for symptomatic patients with HF with reduced ejection fraction (HFrEF) to reduce the risk of HF hospitalization and death.
- An Angiotensin II Receptor Blocker (ARB) is recommended to reduce the risk of HF hospitalization and cardiovascular death in symptomatic patients unable to tolerate an ACE-I (patients should also receive a beta-blocker and a Mineralocorticoid Receptor Antagonist (MRA)).
- Sacubitril/valsartan is recommended as a replacement for an ACE-I to further reduce the risk of HF hospitalization and death in ambulatory patients with HFrEF who remain symptomatic despite optimal treatment with an ACE-I, a beta-blocker and an MRA.
- An MRA is recommended for patients with HFrEF who remain symptomatic despite treatment with an ACE-I and a beta-blocker to reduce the risk of HF hospitalization and death.
- Patients should preferably be enrolled in a disease management program; follow-up plans must be in place prior to discharge and clearly communicated to the primary care team. Patients should be reviewed by their general practitioner within 1 week of discharge and seen by the hospital cardiology team within 2 weeks of discharge if feasible.
- Counseling and treatment for smoking cessation and alcohol intake reduction is recommended for people who smoke or who consume excess alcohol in order to prevent or delay the onset of HF.

In the latest published ESC guidelines from 2021, the diagnostic work-up has been simplified. The new algorithm recommends measuring natriuretic peptides, troponin, creatinine, and electrolytes in all patients, while other blood tests and biomarkers are recommended based on specific clinical scenarios [14,15]. Additionally, the recommendation for routine chest X-rays has been downgraded from Class I to Class IIB (“may be considered”) [14]. Pharmacological therapy recommendations for Class I remain unchanged, with intravenous loop diuretics advised for patients with fluid overload to improve symptoms [15]. Discharge planning has been significantly altered with the evolved management of chronic heart failure, including the introduction of new therapeutic approaches such as SGLT-2 inhibitors and the widespread use of mineralocorticoid receptor antagonists in all patients with HFrEF [15].

## 3. Results

**Figure 1:**
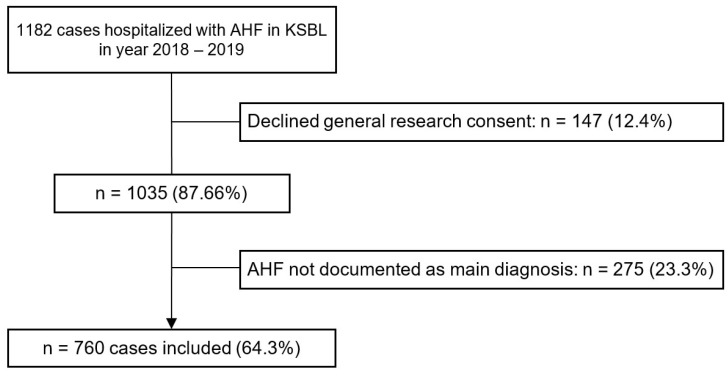
Flowchart of the study enrolment process. Abbreviations: AHF—Acute Heart Failure, KSBL—Cantonal Hospital Baselland.

In total, 1182 cases with the ICD-10 codes specified in Table S1 were extracted from the hospital database for the study. Of these, 147 cases were excluded due to declined general research consent, and 275 cases were excluded because the presentation was not due to AHF as a main diagnosis. This resulted in a study sample of 760 cases. The enrolment process with exclusion criteria and respective number of cases is presented in Figure 1.

### 3.1. Baseline Characteristics

In total, 760 cases were included in the study. The median age of the patients was 84 years, ranging from 45 to 101 years, and 50.3% of the cohort were female. The median body mass index was 26.8 kg/m^2^, with almost one third of the patients being classified as obese (29.9%). Current smokers comprised 10% of the sample and an additional 13.4% were former smokers. In 7.2% of the cases, patients indicated regular alcohol consumption. However, the smoking status was not documented in 59.7% and alcohol consumption in 87% cases. Previous hospitalizations for HF were recorded in 25.4% of cases, and 61.1% of these had occurred within the past 12 months. Detailed baseline characteristics are presented in Table 1.

In our study, patient-reported symptoms at admission varied in prevalence. In the majority of the cases, patients presented with dyspnea (87.2%), followed by weight gain (29.6%), fatigue (20.0%), and orthopnea (18.6%). Less common symptoms included confusion in 2.4%, while nocturnal cough was reported in 2.1%. A group of 3.3% admitted to the emergency department exhibited no typical clinical signs of AHF.

The severity of heart failure according to the New York Heart Association (NYHA) classification was assessed and documented in 38.4% of the cohort, with 30.7% of these cases being classified as NYHA III or IV at the time of admission. Hypertension was identified as the most common etiology for AHF among cases in this study (34.7%), followed by valvular heart disease (28.6%) and arrhythmia (27.1%). In 8.7% of the cases, the exact etiology remained unclear. A cardiomyopathic origin was documented in 5.1% of the cases and the etiology remained unspecified in 3.8% of the cohort. In nearly one-fifth of the cases, a new onset of AHF was reported (18.3%). Detailed symptoms and NYHA classification are shown in Table 2.

The most common comorbidities included hypertension (78.0%), atrial fibrillation (60.3%), and chronic kidney disease (61.2%). Notably, in 32.4% and 15.1% of the cases, diabetes mellitus and a history of cancer, respectively, were documented as comorbidities.

Medication upon admission contained loop diuretics in 76.4% of cases and beta-blockers in 68.3% of cases. In more than half of the cases (54.7%), a medication with ACE-I, ARB or ARNI was recorded as part of the medication prior to admission. A combination of ACE-I, ARB or ARNI and beta-blockers had been prescribed in 39.0% of the cases. The medical history and comorbidities are presented in Table 3.

### 3.2. Diagnostics

**Figure 2:**
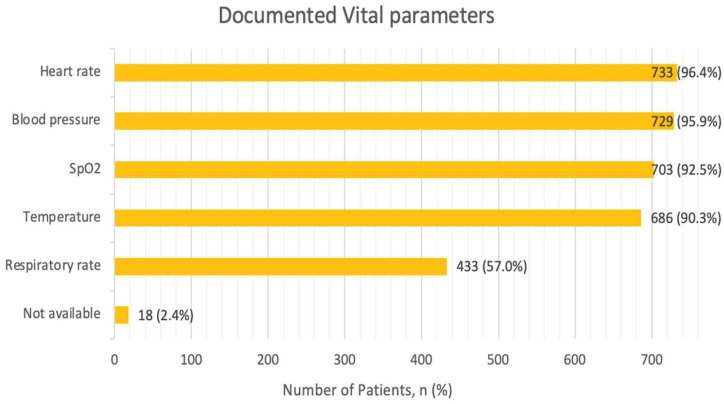
Vital parameters. Multiple parameters per patient possible. Abbreviations: SpO2—Peripheral Capillary Oxygen Saturation.

All the recommended vital parameters, including heart rate, rhythm, respiratory rate, oxygen saturation and blood pressure, were assessed in 51.2% of all cases. Heart rate as well as blood pressure were the most frequently assessed and documented vital parameters, in nearly the entirety of the cohort with 96.4% and 95.9%, respectively (Figure 2). Oxygen saturation was assessed in 92.6%, and body temperature measurements were captured in 90.3% of the cases. Respiratory rate was documented in 56.9% of cases. Vital parameters were not documented in 2.4% of the cases.

The average systolic and diastolic blood pressure were within the normal range with 136.01 (± 26) mmHg and 80.22 (± 17.24) mmHg, respectively (Table S2). Hypertension was noted in 40.3% of cases, while 10.4% were hypotensive with systolic blood pressure below 100 mmHg. The average heart rate was 87.33 (±23.0) bpm, with tachycardia identified in 26.7% and bradycardia in 11.6% of cases. Oxygen saturation levels averaged at 92.91% (±5.1), which included values of 204 cases (26.8%), in which measurements were taken under supplementary nasal oxygen. In the cases where respiratory rate was documented, 29.5% were normopneic (rates < 20 per minute), while 27.5% were tachypneic. Lastly, body temperature readings indicated that patients were afebrile in the majority of cases (91.8%).

**Figure 3:**
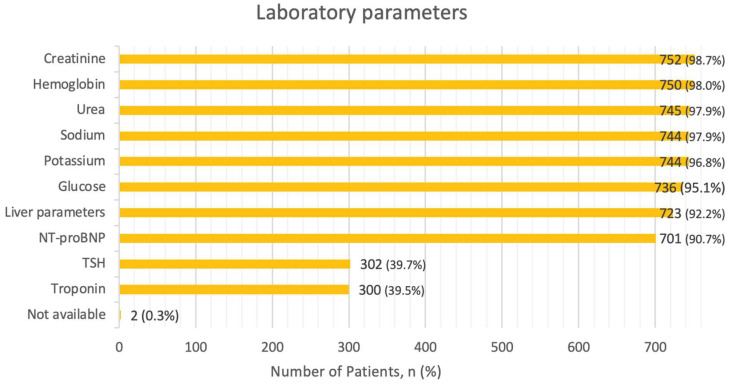
Laboratory parameters. Abbreviations:NT-proBNP—N-terminal pro b-type Natriuretic Peptide, TSH—Thyroid Stimulating Hormone.

Regarding laboratory parameters, in only 16.8% of cases all recommended tests (NT-proBNP, troponin, BUN or urea, creatinine, electrolytes, glucose, blood count, liver parameters, and TSH) were assessed. Creatinine and hemoglobin were the most frequently assessed parameters (98.9% and 98.7%, respectively). Electrolytes potassium and sodium were determined in 97.9% of the cases. Urea levels were checked in 98.0%, blood glucose levels were determined in 96.8%, and liver parameters were obtained in 95.1% of the cases. The NT-proBNP level was obtained in 92.2%, with a median value at admission of 5548.5 ng/L (IQR: 2590.3–13,613 ng/L). TSH and troponin levels were measured in 39.7% and 39.5% of the cases, respectively. The recommended laboratory analyses were not performed in 0.3% of the cases. Percentages of assessed laboratory parameters are presented in Figure 3.

At the time of admission, electrocardiography was performed on the vast majority (95.3%) and chest X-rays were obtained in 89.9% of the patients (Table S3). Echocardiography was carried out for 53.9% of the study participants. Thorax-CT was performed in 5.1% of the study cohort. Coronary angiography was performed in 2.1% of the cases, while cardiac computed tomography and transesophageal echocardiography were undertaken almost equally frequently (1.8% and 1.7%, respectively). Lastly, cardiac magnetic resonance imaging was performed in just 0.4% of the sample.

In the group of 139 cases with de novo AHF and unknown cardiac structure and function, an echocardiography was performed in 84 cases (62.7%). In 32 cases (23%), the echocardiography was conducted within 48 h.

### 3.3. Therapy

Intravenous loop diuretics, specifically furosemide, were administered as recommended in 76.3% of the patients, with an average time to administration of 113.9 min (range: 5 to 392 min). In this group, the time to furosemide treatment was documented in 60%, while thiazide or thiazide-like diuretics were administered in 14.7% of the cases. Opiates for dyspnea relief were given in 3.9%, while digoxin and amiodarone were administered in 2.2% of the cases. Intravenous vasodilators and vasopressors were used in 0.9% and 0.3% of cases, respectively.

### 3.4. Discharge Planning

At discharge, beta-blockers were prescribed in 85.9% of the 163 cases with HFrEF, and ACE-I or ARBs were given in 68.7%. The combination of both ACE-I/ARB and beta-blockers, excluding cases with an LVEF of ≤35%, was prescribed in 59.1% of cases with HFrEF.

Among 116 cases discharged with HFrEF and an LVEF of ≤35%, 55.2% received MRA. Beta-blockers were prescribed in 85.3%, and ACE-I/ARB/ARNI was prescribed in 89.7%. The combination medication of ACE-I/ARB/ARNI, beta-blockers, and MRA was given in 46.6% of the cases.

Management plans at discharge included cardiologic follow-up recommendations in 26.7% of cases. General care follow-ups were not specified in the discharge plan. Target body weights were specified in 69.7% of the cohort, and regular weight measurements were recommended in 62.6% of the cases. Out of the 66 cases of current smokers, referral for smoking cessation advice was documented in only 3 cases (4.5%), while no recommendations for alcohol reduction were documented.

## 4. Discussion

In this retrospective observational cohort study of patients hospitalized with AHF at a Swiss general teaching hospital during 2018–2019, we found that while vital parameters and laboratory diagnostics were generally well-assessed, there are areas with potential for improvement, particularly in echocardiographic diagnostics, loop diuretic therapy, and in the prescription of MRA for patients with HFrEF and LVEF ≤ 35%.

In our study, the median patient age was 84 years, highlighting a notably older demographic compared to the Latin American registry with a median age of 62 years [16]. This age also exceeds that observed in other studies, such as 78 years in Japan and 80 years in the UK [17,18,19]. Additionally, over half of the patients in our cohort were female (50.3%), while other studies reported a majority of male patients [16,17,18,20,21]. The shift in demographics reflects the observation that Switzerland has one of the highest life expectancies in the world, especially for women.

Current smoking, which is known to be associated with impaired heart function and an increased likelihood of hospitalization for AHF [22], was reported in only 10% of the cases with a documented smoking status, a prevalence substantially lower than the overall reported 25% of the Swiss population [23]. With only 6.8% documented as non-smokers and 59.8% of smoking history data missing, the actual number of smokers in our cohort could be higher. Similarly, the status of alcohol consumption, which can trigger AHF and is associated with toxic cardiomyopathy, was documented in only 13% of our cases. This highlights a need for more thorough documentation of risk factors [13]. In comparison, the Danish Heart Failure Registry documented smoking status for 92% of patients and alcohol consumption for 89% [24].

Our study revealed that in 18.3% of cases, patients were diagnosed with de novo AHF. This is significantly lower compared to Japanese (65.3%), Latin American (39.4%) and Canadian (31.3%) studies [16,17,20]. This discrepancy may be due to the lack of clear differentiation between initial and pre-existing heart failure diagnoses in over half of our cases (56.3%).

In our audit, as well as in others, the most common element in medical history was previous PCI, documented in 24.7% of the cases. For comparison, it was 17.5% in Japan and 20.2% in European registries [17]. Furthermore, the occurrence of past CABG and pacemaker implantation was similar to that in European and Japanese registries [17]. However, in the Latin American registry, there was a notably lower incidence of CABG, which is likely related to the significantly younger study population in that region [16].

Hypertensive heart disease was among the most common comorbidities in our study as well as in all examined registries and audits but was notably more prevalent in our audit. Other comorbidities like atrial fibrillation, valvular diseases, and chronic kidney disease were also more pronounced in our study, indicating a higher degree of multimorbidity in our patient population. In contrast, the prevalence of Diabetes Mellitus was lower compared to other analyzed regions.

The underlying etiologies for AHF in our study were 34.7% hypertensive, 28.6% valvular, 27.1% arrhythmic, and 25.8% ischemic. In contrast, other studies, predominantly reported ischemic and valvular causes, followed by hypertensive [16,17,18,20]. Arrhythmias were also significantly observed in the European registry at 41% [21]. These findings indicate a high prevalence of hypertensive heart disease in our study population. Furthermore, the variability in documenting diagnoses and the presence of multiple etiologies as triggers in some cases might have contributed to discrepancies in the data.

The median length of stay in our study was 8 days, which was almost half as long as in Japan (18 days) [17]. Comparable lengths of stay were observed in Canadian and European registries (7 days) [20,21] and in Latin America and the UK (9 days) [16,18]. This similarity across regions, except for Japan, reflects comparable healthcare practices and patient management strategies.

In our study, the in-hospital mortality rate was 9.5%, which is comparable to the UK audit at 10.1% [18]. It was lower in Japan with 7.7% and Canada with 7.8% but significantly higher in Latin America at 17.9% [16,17,20]. The slightly higher mortality in our study, compared to other developed countries, can be attributed to an older and more multimorbid patient population.

The UK audit showed that cardiac rehabilitation leads to improved outcomes after one year for AHF patients [18]. In our study, patients were referred to rehabilitation in 11.7% of cases. This rate shows notable differences when compared to other studies, with only 2% in Canada and a much higher 29.1% of referrals in Japan [17,20]. These disparities highlight the varying approaches to post-AHF care in different countries.

### 4.1. Diagnostics of AHF

Regarding the documentation of vital parameters in our study, heart rate and blood pressure were measured in almost all cases, at 96.4% and 95.9%, respectively, followed by oxygen saturation in 92.5% of cases. This demonstrated very good compliance with guideline recommendations. The respiratory rate, however, was measured in just over half the cases, amounting to 57%. This trend was similar to the Canadian registry, which showed comparable results for heart rate (96.9%) and blood pressure (96.6%) measurements [20]. Notably, none of the studied registries emphasized the measurement of respiratory rate. Generally, the importance of assessing respiratory rate in AHF patients is partly neglected, even though studies suggest that this parameter, in conjunction with oxygen saturation or heart rate, can serve as a predictor of mortality and ICU admissions [25].

Remarkably, the measurement of NT-proBNP upon presentation was recorded in our audit population in 92.2% of cases, significantly exceeding the 34.7% in the European and 26.9% in the Canadian registries [20,21]. The lower rate in Canada is likely due to limited availability of devices to test or cost reasons, despite local guideline recommendations [26]. This highlights the importance of NT-proBNP in heart failure assessment and the variability in its use across different regions. A pilot study in Romania found a correlation between cardiac biomarkers and ECG changes in patients with acute heart failure, which may lead to a more complex predictive model and improve the clinical approach for these patients [27]. Underlining the importance of the cardiac biomarker, other clinical trials investigated the value of serial NT-proBNP measurements for AHF monitoring and therapy guidance [28,29]. However, these trials presented mixed results and did not lead to any global recommendations.

Laboratory tests were generally well conducted, though TSH (39.7%) and troponin (39.5%) testing rates could be improved. In comparison, the European registry had a similar troponin determination rate of 43.8% [21]. A better outcome was observed in the Canadian study, which reported a 60.3% measurement rate for troponin [20]. This highlights the areas where lab testing could be more consistently applied in the future.

In our analysis, the performance of ECG and chest imaging showed very high adherence to guidelines, at 95.3% for ECG and 95% for chest imaging (chest X-ray (89.9%) and thorax-CT (5.1%)). By comparison, the Canadian registry reported ECG in 84.1% and chest X-ray in 82.1% of AHF patients [20]. In the UK audit, ECG was performed in 86% of study participants, but no data regarding chest X-ray was provided, despite NICE guideline recommendations [18]. This reflects a strong compliance with recommended diagnostic procedures in our study. In recent studies, it has been shown that machine learning models using ECG or circadian ECG features could accurately classify patients into groups based on their ejection fraction and identify those with reduced ejection fraction. This could be particularly useful in the management of heart failure patients when echocardiography is not available [30,31,32].

Diagnostics with echocardiography was performed on 53.9% of cases and in over a fifth (22.5%) of all cases, patients had prior echocardiographic findings from the preceding year. Among the 139 cases with de novo AHF, in 62.7%, an echocardiography was performed, and 23% of these were within the first 48 h. Less than half of the 31 cases treated in the ICU received an echocardiogram. This suggests that acute management may lead to delays and possibly omission of recommended, time-consuming procedures. The Canadian registry reported a similar rate of echocardiography at 54%, while the UK audit impressively showed 88% [18,20]. In the UK, this result was potentially reached due to echocardiograms being performed by both clinicians and clinical scientists, easing specialist workload and increasing diagnostic capacity [33,34]. Echocardiography is crucial for determining AHF etiology and guiding appropriate treatment [35]. A non-optimal use of echocardiography was associated with logistical problems and possibly a lack of documentation. With the implementation and support of machine learning-based algorithms, the application of this diagnostic method could be simplified and potentially more widely adopted in clinical practice [36].

### 4.2. Therapy of AHF

In terms of therapy with diuretics, 94.5% of cases presented with at least one clinical sign of volume overload in our emergency department. Intravenous furosemide was initiated in 76.3% of cases, this indicating a gap in guideline adherence. Comparable use of intravenous diuretic medication was observed in the European registry at 75.6% and in the Japanese registry at 81.7% [17]. However, these registries did not specify the number of patients presenting with signs of hypervolemia. This highlights a potential area for improved adherence to guidelines in the management of volume overload in AHF patients. Diuretics have been proven to relieve symptoms and improve exercise capacity in patients with heart failure [37]. Early administration of intravenous loop diuretics is associated with reduced in-hospital mortality as well [38].

### 4.3. Discharge Planning

In the sub-group of 163 HFrEF cases, beta-blockers were given in 85.9% and ACE-I/ARB in 68.7%. In the Canadian registry, ACE-I/ARB administration was at 56.8% and beta-blockers at 80.4% [20]. In the UK, 84% were treated with ACE-I/ARB and 89% with beta-blockers [18]. This highlights regional variations in guideline compliance, particularly for beta-blockers, where our hospital could improve.

As part of the hospital discharge planning among the 116 HFrEF cases with LVEF ≤ 35%, MRA were prescribed in 55.2%. Regarding the guideline recommendations, this has ample potential for improvement. In European registries, MRA was given to 53.9% of all discharged patients, although without specific echocardiographic subgrouping [21]. The guidelines in Canada and the UK recommend initiating MRA in patients of HFrEF, so this data could not be directly compared [26]. This highlights regional differences and also highlights the need for further research in this specific patient group. MRA lowers the risk of morbidity and mortality in patients with severe heart failure [39].

Upon discharge, HF patients are typically referred to an outpatient physiotherapy program. Furthermore, regular weight monitoring is essential for ongoing therapeutic adjustment. In our study, the discharge weight and target weight were recorded in 62.6% and 69.7% of cases, respectively. There is room for improvement in our discharge procedures.

Regarding follow-up recommendations, a prompt consultation with the treating primary care physician is recommended for all hospitalized patients. While the assessment of primary care follow-up recommendations was not possible due to lack of documentation, cardiologist follow-up was recommended in 26.7% of cases. By comparison, in the Canadian registry, 51% of patients received specialist follow-up [20]. Recent studies have shown that patients with cardiovascular diseases are at increased risk of early readmission [40]. The development of scoring systems could facilitate tailored treatment for patients with AHF, help predict the short-term risk of readmission or death, and thereby improve the post-discharge management of AHF patients [41]. Furthermore, with continued technological advancements and the expansion of telemedicine services, post-discharge care could be significantly improved in the future [42]. This could lead to reduced outpatient visits, hospitalizations, and lengths of hospital stays, while also contributing to the optimization of healthcare resources [42]. The study by Trimarchi et al. highlights the importance of identifying etiology-specific predictors of ventricular function recovery, enabling tailored medical therapy to improve left ventricular (LV) function in patients with heart failure [43].

Smoking cessation advice was recommended in only 4.5% of the active smokers upon discharge. Additionally, no recommendations were documented regarding alcohol intake reduction. This indicates that the awareness of risk factors and their management needs significant improvement. Furthermore, noncompliance to smoking and alcohol restrictions significantly elevates the risk for hospital readmissions [44].

### 4.4. Study Limitations

In our audit, several limitations were identified. Firstly, the data were collected retrospectively, which could result in underestimating outcomes due to potentially incomplete patient records. Additionally, while some data were automatically sourced from the patient management system, the majority required manual extraction from various patient documents. Despite multiple checks, this process may cause errors in data collection. Only a few audits and registries on AHF management were available for comparison, often focusing on specific aspects of AHF management. Our study covered a broader range of variables. However, some data could not be directly compared with other studies due to differences in local guideline recommendations.

## 5. Conclusions

This audit, a part of the “QUA-DIT” (Quality Evaluation of Hospital Care Through Audits) project, demonstrated generally good adherence to AHF guideline recommendations, though several areas require improvement. Particularly, assessment and documentation of weight, NYHA classifications, smoking status, respiratory rate, and oxygen therapy were suboptimal. Improvements are also needed in the laboratory-based diagnostic assessment of troponin and TSH and in performing echocardiography in patients with de novo AHF. Furthermore, initiating intravenous diuretic medication in patients showing clinical signs of hypervolemia and planning of the discharge are areas that could be improved.

Compared to other AHF audits and registries, our study’s population was older, included a slightly higher proportion of females, had a greater prevalence of pre-existing hypertensive heart disease, and presented themselves more often with acute decompensated HF. Diagnostic workup for AHF using ECG, chest imaging, and laboratory determination of natriuretic peptides were conducted more adherent with guidelines than in other studies. The use of intravenous diuretics was comparable to other settings. Additionally, the duration of hospitalization and mortality rates, despite regional differences, were similar to other analyzed studies [16,17,18,20,21]. Since results were comparable to other audits, we hypothesize that these findings might be transferrable to other European hospitals to a certain extent.

The QUA-DIT project aims to enhance hospital care by evaluating adherence to disease-specific clinical guidelines across key areas in internal medicine, ultimately seeking to improve patient outcomes through comprehensive quality control programs [45]. In this context, the results of this audit will contribute to the efficiency of AHF management and will help to improve outcomes for AHF patients. To ensure continued high-quality AHF management and develop further enhancement, regular implementation of such audits should be established in the future.

## Figures and Tables

**Table 1:** Baseline characteristics.

	All (*n* = 760)	Missing, *n* (%)
Weight, median (IQR) in kg	74.8 (63.3–86.9)	293 (38.6)
Body mass index, median (IQR) in kg/m^2^	26.8 (23.3–30.8)	362 (47.6)
Obesity, BMI ≥ 30, *n* (%)	175 (23)	362
Smoking status		530 (59.7)
Current smoker, *n* (%)	76 (10.0)	
Former smoker, *n* (%)	102 (13.4)	
Non-smoker, *n* (%)	52 (6.8)	
Alcohol consumption		661 (87.0)
Excessive, *n* (%)	30 (3.9)	
Moderate, *n* (%)	25 (3.3)	
No alcohol, *n* (%)	44 (5.8)	
Assignment via		
Family doctor, *n* (%)	263 (34.6)	
Self-Assignment, *n* (%)	246 (32.4)	
Ambulance, *n* (%)	183 (24.1)	
Other, *n* (%)	68 (8.9)	
Previous hospitalization due to heart failure	193 (25.4)	
Hospitalization because of AHF within last 12 months	118 (61.1)	
De novo AHF	139 (18.3)	
Admission to the ICU, *n* (%)	31 (4.1)	
Length of stay, median (IQR) in days	8 (5 to 12)	
In-hospital death, *n* (%)	72 (9.5)	
Residence after discharge		
Home, *n* (%)	583 (76.7)	
Rehabilitation, *n* (%)	89 (11.7)	
Other hospital, *n* (%)	13 (1.7)	
Hospice, *n* (%)	3 (0.4)	

Abbreviations: ICU—Intensive Care Unit; IQR—Interquartile Range.

**Table 2:** Symptoms and NYHA classification.

	All (*n* = 760)	Missing, *n* (%)
Symptoms at admission		
Dyspnoe, *n* (%)	663 (87.2)	
Weight increase, *n* (%)	225 (29.6)	
Fatigue, *n* (%)	152 (20.0)	
Orthopnea, *n* (%)	141 (18.6)	
Angina pectoris, *n* (%)	76 (10)	
Confusion, *n* (%)	18 (2.4)	
Nocturnal cough, *n* (%)	16 (2.1)	
No symptoms, *n* (%)	22 (3.3)	
NYHA classification		468 (61.6)
NYHA I, *n* (%)	5 (0.7)	
NYHA II, *n* (%)	54 (7.1)	
NYHA III, *n* (%)	104 (13.7)	
NYHA IV, *n* (%)	129 (17.0)	

Abbreviations: NYHA—New York Heart Association.

**Table 3:** Medical history and comorbidities.

	All (*n* = 760)	Missing, *n* (%)
Medical history		
Previous PCI, *n* (%)	188 (24.7)	
Previous MI, *n* (%)	134 (17.6)	
Pacemaker, *n* (%)	79 (10.4)	
Previous stroke/TIA, *n* (%)	69 (9.1)	
Previous CABG, *n* (%)	68 (8.9)	
Previous valvular surgery, *n* (%)	51 (6.7)	
ICD, *n* (%)	26 (3.4)	
CRT, *n* (%)	9 (1.2)	
Other, *n* (%)	10 (1.3)	
Comorbidities		
Hypertension, *n* (%)	593 (78.0)	
Atrial fibrillation, *n* (%)	458 (60.3)	
Valvular heart disease, *n* (%)	351 (46.2)	
Coronary artery disease, *n* (%)	285 (37.5)	
Chronic kidney disease, *n* (%)	465 (61.2)	
Anemia, *n* (%)	250 (32.9)	
Diabetes mellitus, *n* (%)	246 (32.4)	
Cancer, *n* (%)	115 (15.1)	
Active Cancer, *n* (%)	47 (40.9)	
Hypothyroidism, *n* (%)	107 (14.4)	
COPD, *n* (%)	106 (13.9)	
Pulmonary hypertension, *n* (%)	50 (6.6)	
Asthma, *n* (%)	24 (3.2)	
Peripheral artery disease, *n* (%)	88 (11.6)	
Sleep apnea syndrome, *n* (%)	78 (10.3)	
Depression, *n* (%)	46 (6.1)	
Hyperthyroidism, *n* (%)	26 (3.4)	
Other arrhythmia, *n* (%)	19 (2.5)	
Medication upon admission		41 (5.4)
ACE-Inhibitor, *n* (%)	235 (32.7)	
ARB, *n* (%)	135 (18.8)	
ARNI, *n* (%)	23 (3.2)	
Beta-blocker, *n* (%)	491 (68.3)	
Loop diuretics, *n* (%)	549 (76.4)	
Mineralocorticoid receptor antagonists, *n* (%)	104 (14.5)	
Thiazide diuretic, *n* (%)	117 (16.3)	
None, *n* (%)	66 (9.2)	
Combined medication upon admission		
ACE-I/ARB/ARNI and beta-Blocker, *n* (%)	31 (4.3)	
ACE-I/ARB/ARNI, beta-Blocker and loop diuretics, *n* (%)	200 (27.8)	
ACE-I/ARB/ARNI, beta-Blocker and MRA, *n* (%)	4 (0.6)	
ACE-I/ARB/ARNI, beta-Blocker, MRA and loop diuretics, *n* (%)	45 (6.3)	

Abbreviations: ACE-Inhibitor—Angiotensin-Converting Enzyme Inhibitor, ARB—Angiotensin II Receptor Blocker, ARNI—Angiotensin Receptor-Neprilysin Inhibitor, CABG—Coronary Artery Bypass Grafting, COPD—Chronic Obstructive Pulmonary Disease, MI—Myocardial Infarction, MRA—Mineralocorticoid Receptor Antagonist, PCI—Percutaneous Coronary Intervention, TIA—Transient Ischemic Attack.

## Data Availability

The data presented in this study are available from the corresponding author upon reasonable request. The data are not publicly available due to restrictions in data privacy.

## Supplementary Materials

The following supporting information can be downloaded at: https://www.mdpi.com/article/10.3390/jcm13195951/s1, Table S1: ICD-10 codes; Table S2: Vital parameters at time of admission; Table S3: Diagnostic methods.
