# Supplementary material for: Diagnosis and Treatment of Acute Heart Failure: A Retrospective Observational Study and Medical Audit
Source: J Clin Med. 2024 Oct 7;13(19):5951. doi: 10.3390/jcm13195951 (PMC11478339; doi:10.3390/jcm13195951)
Supplement: Supplementary file 1 [file jcm-13-05951-s001.zip › jcm-3205867-supplementary.pdf]

Supplementary Materials

Table S1. ICD-10 codes.

**I11.0: Hypertensive heart disease with (congestive) heart failure**
I13.0: Hypertensive heart and kidney disease with (congestive) heart failure.
I50.0: Congestive heart failure
I50.1: Left ventricular failure
I50.2: Systolic heart failure
I50.3: Diastolic heart failure
I50.4: Combined systolic and diastolic heart failure
I50.8: Other heart failure
I50.9: Unspecified heart failure

Table S2. Vital parameters at time of admission.

	**All (n = 760)**	**Missin n (%)**
Heart rate, mean (SD) in bpm	87.33 (±23.0)	27 (3.6)
Tachycardia (≥ 100 bpm), n (%)	196 (26.7)	
Bradycardia (< 60 bpm), n (%)	85 (11.6)	
Blood pressure mean (SD)		31 (4.1)
Systolic blood pressure (mmHg), mean (SD)	136.01 (± 26.26)	
Diastolic blood pressure (mmHg), mean (SD)	80.22 (± 17.24)	
Hypertension (systolic blood pressure >140 mmHg), n (%)	294 (40.3)	
Hypotension (systolic blood pressure < 100 mmHg), n (%)	45 (6.17)	
Oxygen saturation ± oxygen therapy, mean (SD) in %	92.91 (±5.1)	57 (7.5)
Nasal oxygen therapy, n (%)	204 (26.8)	
Body temperature °C, mean (SD)		74 (9.7)
Afebrile (< 37.0° C), n (%)	630 (91.8)	
Subfebrile (37.1-37.9°C), n (%)	43 (6.3)	
Febrile (≥ 38.0°C), n (%)	13 (1.9)	
Respiratory rate		327 (43.0)
Normopnea (<20/min)	224 (29.5)	
Tachypnea (>20/min)	209 (27.5)	

Abbreviations: bpm - Beats per minute, mmHg – Millimeters of mercury, SD – standard deviation.

Table S3. Diagnostic methods.

	**n = 760**
ECG, n (%)	724 (95.3)
Chest X-ray, n (%)	683 (89.9)
Echocardiography	410 (53.9)
Echocardiography prefindings from the preceding year	171 (22.5)
Thorax-CT, n (%)	39 (5.1)
Coronary angiography, n (%)	16 (2.1)
Cardiac computed tomography, n (%)	14 (1.8)
Transoesophageal echocardiography, n (%)	13 (1.7)
Cardiac magnetic resonance, n (%)	3 (0.4)

Abbreviations: ECG - Electrocardiogram.
